# An investigation into the effects of cow genetic background and pasture-based management system on circulating pregnancy-associated glycoprotein concentrations during early pregnancy

**DOI:** 10.3168/jdsc.2025-0997

**Published:** 2026-04-23

**Authors:** R.C. Doyle, J. Kenneally, M.C. Lucy, S.T. Butler

**Affiliations:** 1 Teagasc, Animal & Grassland Research and Innovation Centre, Moorepark, Fermoy, Co. Cork, Ireland P61 P302; 2 Department of Animal Science, University of Missouri, Columbia, MO 65201

## Abstract

Cows with greater circulating pregnancy-associated glycoprotein (PAG) concentrations after artificial insemination (AI) are more likely to maintain pregnancy (i.e., less pregnancy loss). The objective was to evaluate the effect of cow genetic background and feeding level on circulating PAG during early pregnancy in seasonal-calving, pasture-based, lactating dairy cows. Three divergent genetic groups (GG) of dairy cows were enrolled: Elite Economic Breeding index (EBI) Holstein-Friesian cows (Elite), representative of the top 5% nationally; average EBI Holstein-Friesian cows (NA), representative of the national average in Ireland; and purebred Jersey (JE) cows, representative of Jersey genetics available in Ireland and used primarily for crossbreeding. The 3 GG were evaluated under 3 contrasting spring-calving pasture-based feeding treatments (FT): control (CTL), low grass allowance (LGA), and high concentrate (HC). A total of 108 cows (36 in each GG and each FT) were initially enrolled. Cows were artificially inseminated following detected estrus at the start of the seasonal breeding period, and if cows did not return to estrus, blood samples were collected on d 23, 25, and 28 after AI (n = 92 samples collected; 16 cows returned to estrus before d 23). Ultrasound examinations for pregnancy diagnosis were conducted on d 30 to 36 after AI (hereafter referred to as d 33); if pregnant, cows were re-examined on d 60 to 66 after AI (hereafter referred to as d 63). Plasma PAG concentrations were measured using an IDEXX kit, and pooled blood from Holstein-Friesian cows that were on d 35 ± 2.4 (mean ± SD) of gestation was used to create a standard curve. Blood PAG concentrations were expressed relative to the concentration in the pooled sample from pregnant cows (% PC). There was evidence of an effect of GG on the PAG profile observed during early gestation. Jersey (n = 21) cows had greater mean (95% CI) plasma PAG concentrations compared with Elite (n = 20) cows but not NA (n = 13) cows: 52.1% PC (39.5%, 66.8%), 32.0% PC (22.5%, 43.5%), and 36.3% PC (24.3%, 51.4%), respectively. There was no effect of FT on circulating PAG concentrations. Across all cows sampled, circulating PAG concentrations were greater in cows that subsequently maintained their pregnancy (n = 54) compared with cows that subsequently had pregnancy loss (n = 9), and concentrations were lowest in cows that had no evidence of pregnancy based on plasma PAG measurements in advance of ultrasound diagnosis on d 33 (n = 29). Overall, the study findings indicate that cow genetic background and feeding treatment had little effect on circulating PAG concentrations during early pregnancy in grazing dairy cows. Pregnancy-associated glycoprotein determination on d 23 to 28 after AI could aid identification of pregnancy status and future risk of pregnancy loss.

## Highlights

• Jersey cows had higher early pregnancy PAG concentrations than Holstein-Friesians.
• Pregnancy-associated glycoprotein concentrations were similar in the 2 strains of Holstein-Friesian cows.
• There was no evidence that nutritional management affected circulating PAG concentrations.
• Pregnancy-associated glycoprotein concentrations < day 28 after AI may identify pregnancies at risk of loss.
• Pregnancy-associated glycoprotein concentrations < day 28 after AI may identify nonpregnant cows.

## Graphical Abstract

We investigated the effect of cow genetic background and nutritional management on circulating pregnancy associated glycoprotein (PAG) concentrations during early gestation (days 23, 25, and 28 after artificial insemination [AI]). Of the 108 cows enrolled, 16 returned to estrus before day 23. Blood samples were collected from the remaining 92 cows. Circulating PAG concentrations were greater in Jersey than Holstein-Friesian cows and were unaffected by feed treatment. Among cows that had not returned to estrus, PAG concentrations were greater in cows that maintained pregnancy (n = 54) versus cows that experienced pregnancy loss between day 33 and 63 after insemination (n = 9) or never had evidence of being pregnant based on circulating PAG concentration before ultrasound pregnancy diagnosis on day 33 (n = 29). Measuring PAG concentration before day 28 after AI may help identify nonpregnant cows and pregnancies at risk of loss. HC = high concentrate; JE = Jersey; LGA = low grass allowance; NA = average Economic Breeding index Holstein-Friesian cows. Created with BioRender.com.

**Figure:**
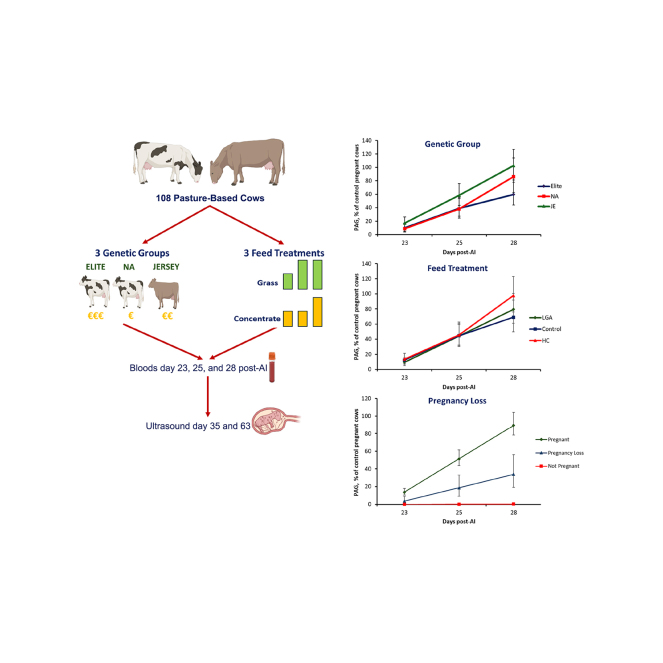

Pregnancy-associated glycoproteins (**PAG**) are produced by trophoblast giant cells of the developing embryo (Xie et al., 1991; Zoli et al., 1992a; Green et al., 2000). The trophoblast giant cells migrate and fuse with the epithelial cell lining of the uterus at the time of implantation around d 19 of gestation (Boshier, 1969; Guillomot, 1995), releasing their contents into the capillary bed of the uterus that connects to maternal peripheral circulation (Spencer et al., 2007; Lucy et al., 2011). The placenta produces more than 20 closely related PAG proteins (Telugu et al., 2009). Pregnancy-associated glycoproteins can be detected as early as 25 to 28 d after AI in blood and milk of pregnant cows (Zoli et al., 1992b; Green et al., 2005; Friedrich and Holtz, 2010). Moreover, recent studies demonstrated that daily measurement of serum concentrations of Pregnancy Specific Protein-B (a member of the PAG family) from d 19 to 28 after artificial insemination (**AI**) could identify nonpregnant cows, cows with early conceptus attachment (d 19 or 20), and cows with late conceptus attachment (≥ d 21). Cows with late conceptus attachment had markedly greater risk of later pregnancy loss (Santos et al., 2023; Crowe et al., 2025a).

Concentrations of PAG are 2-fold greater in plasma compared with milk (Fricke et al., 2015; Ricci et al., 2015), and measurements in both matrices have been validated for early pregnancy diagnosis in cattle. Plasma PAG concentrations at d 30 of gestation have been suggested as potential markers for embryo mortality or viability between d 30 and 60 of gestation (Gábor et al., 2007; Pohler et al., 2016a; Ergene et al., 2019), but the prediction accuracy is antibody dependent (Gatea et al., 2018). Greater circulating PAG concentrations may therefore indicate a more functional placenta with a greater potential to establish and maintain pregnancy.

Selection indices have undergone substantial changes in the past 25 years (Lucy, 2019). Current multitrait selection indices include fertility, health and fitness traits (Miglior et al., 2017; Cole and VanRaden, 2018). The Economic Breeding Index (**EBI**) is a multitrait selection index to identify the most profitable sires and dams to breed replacement animals for Irish dairy production systems (Berry et al., 2005). A controlled multiyear study involving “The Next Generation Herd” reported that Elite EBI cows (top fifth percentile of national herd based on EBI) had greater milk solids yield (O'Sullivan et al., 2019b), improved reproductive efficiency (O'Sullivan et al., 2020a) and greater profitability (O'Sullivan et al., 2020b) compared with national average (**NA**) EBI cows (representative of the national average EBI). Previously, with the same herd, we reported improved postpartum reproductive tract health status and greater luteal phase plasma progesterone concentrations in Elite versus NA and Jersey (**JE**) cows (Doyle et al., 2026). The objective of the current study was to test the effect of cow genetic background and feed treatment on plasma PAG concentrations in lactating dairy cows managed under a seasonal-calving pasture-based production system. We tested the hypothesis that plasma PAG concentrations during the early period of conceptus attachment and placental development are influenced by dam genetic background and feed treatment during early gestation.

This study was carried out at the Teagasc Dairygold Research Farm (County Cork, Ireland) in May 2018 (52°09′N; 8°16′W). Lactating dairy cows (n = 108) were managed in a typical spring-calving, pasture-based production system, as described previously (O'Sullivan et al., 2019b; Doyle et al., 2026). Genetic groups were balanced for parity and calving date each year. Briefly, 36 Elite EBI Holstein-Friesian (**HF**) cows (**Elite**; within the top fifth percentile of cows nationally ranked on EBI), 36 NA EBI cows (representative of national average genetic merit based on EBI) and 36 purebred JE cows representative of Jersey genetics available in Ireland (originating from New Zealand and Denmark) and used primarily for crossbreeding were enrolled in the experiment. Information on cows enrolled in the study, including EBI values, individual subindex values, and PTA values for individual genetic traits, was sourced from the Irish Cattle Breeding Federation national database (https://www.icbf.com/) in 2018 and is summarized for the Elite, NA, and JE genetic groups (**GG**) in Table 1.

**Table 1:** The mean Economic Breeding Index (EBI), EBI subindices, and PTA values for selected individual traits for the 3 genetic groups of cows studied (SD in parentheses)^1^

Category	Elite (n = 36)	NA (n = 36)	Jersey (n = 36)
EBI (€)	192.2 (29.1)	78.2 (34.1)	101.2 (43.1)
EBI subindex			
Milk (€)	35.3 (18.2)	24.0 (22.3)	16.6 (24.5)
Fertility (€)	116.5 (22.7)	30.2 (23.3)	48.7 (18.6)
Calving (€)	35.8 (9.6)	30.7 (10.1)	11.80 (21.3)
Beef (€)	−13.60 (8.1)	−9.2 (7.3)	−50.9 (14.5)
Maintenance (€)	14.8 (1.5)	3.8 (9.8)	70.0 (19.7)
Management (€)	1.8 (3.2)	0.5 (3.4)	−0.40 (2.6)
Health (€)	1.5 (3.5)	−0.2 (5.0)	5.5 (2.8)
PTA			
Calving interval (d)	−6.1 (1.3)	−1.70 (0.8)	−1.8 (0.7)
Survival (%)	3.4 (0.7)	1.4 (1.1)	2.2 (0.9)
Milk (kg)	−37.8 (145.6)	30.1 (122)	−486 (146.9)
Fat (kg)	7.5 (5.0)	3.88 (4.1)	8.26 (5.7)
Protein (kg)	3.64 (3.7)	2.42 (3.8)	4.98 (4.0)
Fat (%)	0.16 (0.11)	0.09 (0.10)	0.50 (0.15)
Protein (%)	0.09 (0.04)	0.061 (0.05)	0.24 (0.07)

1 Elite = high EBI; NA = national average EBI.

Cows within each GG were randomly assigned before calving to 1 of 3 pasture-based experimental feeding treatments (**FT**). Randomization was carried out after blocking the cows based on EBI, parity, calving date, milk solids yield from the previous lactation, BW, and BCS, as previously described (O'Sullivan et al., 2019b). The experimental FT were control (**CTL**), low grass allowance (**LGA**), and high concentrate (**HC**), with target postgrazing sward heights of 4.5 to 5.0 cm, 3.5 to 4.0 cm, and 4.5 to 5.0 cm and a total lactation concentrate intake of 300, 300, and 1,100 kg, respectively (O'Sullivan et al., 2019a). The 3 FT were designed to represent management scenarios reflective of the upper and lower limits of recommended best practice to maximize productivity in Irish milk production systems. Elite, NA, and JE cows within each of the assigned FT were grazed separately in adjacent paddocks. Cows were milked twice daily at 0700 and 1600 h (data not presented here).

Uterine and ovarian statuses were assessed in all cows greater than 30 DIM using transrectal ultrasonography (Ibex Pro scanner with an 8.5 MHz transducer, E.I. Medical Imaging, Loveland, CO) before the mating start date (**MSD**). Cows had tail paint applied twice weekly as part of the normal management practice to aid identification of estrus, starting 3 wk before MSD. Heat detection was carried out a minimum of 3 times daily during the breeding season with the aid of tail paint and activity monitoring collars (MooMonitor, Dairymaster, Causeway, Co. Kerry, Ireland) that had been fitted to each animal before parturition. The breeding season commenced on April 25, 2018, and all cows were inseminated using frozen thawed conventional semen from a team of bulls during the first 6 wk of the breeding season, which encompassed the period of interest for the current study. The same bulls were used for AI on the Elite and NA cows, and purebred JE bulls were used for the JE cows. Pregnancy was diagnosed by transrectal ultrasound at 30 to 36 d after AI (hereafter referred to as d 33). Cows diagnosed pregnant were re-examined again at 60 to 66 d (hereafter referred to as d 63) after AI to confirm pregnancy or detect cows that had pregnancy loss (**PL**). Pregnancy loss was deemed to have occurred if a cow was pregnant at 33 d and subsequently diagnosed nonpregnant at 63 d.

Blood samples were collected on d 23, 25, and 28 after AI into evacuated tubes containing lithium heparin (Becton Dickinson, Plymouth, UK). Samples were centrifuged at 2,000 × *g* for 15 min at 4°C, and the plasma was decanted into individual polypropylene tubes and stored at −20°C. Relative plasma concentrations of PAG were determined (n = 4 assays) using a commercially available ELISA kit (PAG; IDEXX Switzerland AG, Liebefeld-Bern, Switzerland). Two reference samples, one low-PAG (i.e., nonpregnant pooled sample: negative control) and one high-PAG (i.e., pregnant animal pool: positive control) were included in each assay for accuracy assessment and quality control. The commercial ELISA kit used in this study was a qualitative assay that we modified to be semiquantitative, similar to the approach previously described by Fricke et al. (2016). The assays were conducted according to the manufacturer's instructions, with the exception that a standard curve was generated using a pooled sample of plasma collected from pregnant Holstein-Friesian cows (n = 6; mean day of gestation ± SD = 35 ± 2.4, range 32 to 42) that was serially diluted with plasma collected from nonpregnant cows (100%, 50%, 25%, 12.5%, 6.25%, and 0%). Standards, controls, and plasma samples were assayed in duplicate on each plate, and optical density readings were obtained using a microtiter plate reader (ELx808, BioTek, Winooski, VT). The standard curve for each plate was used to calculate the plasma concentrations of PAG expressed as a percentage relative to d 35 pregnant cows (**% PC**). The inter- and intra-assay CV for plasma PAG were 14.8% and 4.9%, respectively.

All statistical analyses were performed using SAS 9.4 software (SAS Institute, Cary, North Carolina). The experimental unit was the individual cow and cow nested within GG was included as a random effect. Before statistical analysis, plasma PAG concentrations were checked for normality using the Shapiro–Wilk statistic and graphical methods obtained with PROC UNIVARIATE of SAS. Pregnancy-associated glycoprotein concentrations were not normally distributed; an appropriate Box–Cox transformation was identified and applied before analysis, and results are presented as back-transformed LSM with 95% CI.

For the initial analysis, only cows that conceived to first AI and were confirmed pregnant on d 63 were included (n = 54). Treatment differences in plasma PAG concentrations for pregnant cows on d 23, 25, and 28 were analyzed using mixed models (PROC MIXED in SAS), and results are presented as back-transformed LSM and 95% CI. To identify factors associated with the relative plasma concentrations of PAG in pregnant cows, a multivariable mixed model was used and included the fixed effects of GG (Elite [n = 20], NA [n = 13], JE [n = 21]), FT (LGA [n = 20], CTL [n = 14], HC [n = 20]), days after AI (23, 25, 28), parity (1 [n = 19], 2 [n = 17], ≥3 [n = 18]), and 2-way interactions between GG, FT, and day. Days after AI was treated as a repeated measure with a compound symmetry covariance structure. Denominator degrees of freedom were estimated using the Kenward–Roger method. Contrast statements were constructed to compare GG and FT within each individual blood sample collection day after AI (23, 35, and 28), and to compare JE and HF (i.e., mean of Elite and NA). Pairwise comparisons among LSM were conducted using Tukey adjustment to account for multiple comparisons.

In a second analysis using the full dataset (all cows that were blood sampled), cows were classified into the following pregnancy status groups: conceived to first AI and maintained pregnancy (**PREG**; n = 54); no evidence of pregnancy based on plasma PAG measurements in advance of ultrasound diagnosis on d 33 (**NOT PREG**; n = 29 [Elite = 8, NA = 9, JE = 12; LGA = 9, CTL = 12, HC = 8]); and cows that conceived to first AI but later had pregnancy loss between d 35 and d 63 (PL; n = 9 [Elite = 4, NA = 3, JE = 2; LGA = 4, CTL = 2, HC = 3]). The association between cow pregnancy status group and circulating PAG concentrations was analyzed using mixed models (PROC MIXED). The model included the fixed effects of cow pregnancy status group (PREG, NOT PREG, PL), days after AI (23, 25, 28), parity, DIM at AI, and 2-way interactions between cow pregnancy status group and days after AI. Cow nested within GG was included as a random effect, and days after AI was modeled as a repeated measure using a compound symmetry covariance structure. Degrees of freedom were approximated using the Kenward–Roger method. Contrast statements were constructed to compare pregnancy status groups within each individual blood sample collection day after AI.

We found no interaction between GG and FT for circulating plasma PAG concentrations (*P* = 0.85). Within GG, JE cows tended to have greater (*P* = 0.07) mean circulating plasma PAG concentrations [52.1% PC, (39.5%, 66.8%)] compared with Elite [32.0% PC, (22.5%, 43.5%)] and NA [36.3% PC, (24.3%, 51.4%)] cows. There was evidence of an interaction between GG and day after AI for circulating PAG concentrations (*P* = 0.07; Figure 1A). This interaction arose because JE cows had greater plasma PAG concentrations on d 25 compared with both Elite cows (*P* = 0.08) and NA cows (*P* = 0.09). On d 28, plasma PAG concentrations were greater in both JE (*P* = 0.04) and NA (*P* = 0.08) cows than Elite cows. When breeds were compared, JE cows had greater (*P* = 0.03) mean circulating plasma PAG concentrations (51.0% PC [39.1%, 64.8%]) compared with HF cows (34.3% PC [26.9%, 42.9%]). We found no breed by day interaction effect (*P* = 0.82) on plasma PAG concentrations. No effect of FT (*P* = 0.64) or FT by day interaction was observed (*P* = 0.15; Figure 1B) on mean circulating plasma PAG concentrations.

**Figure 1:**
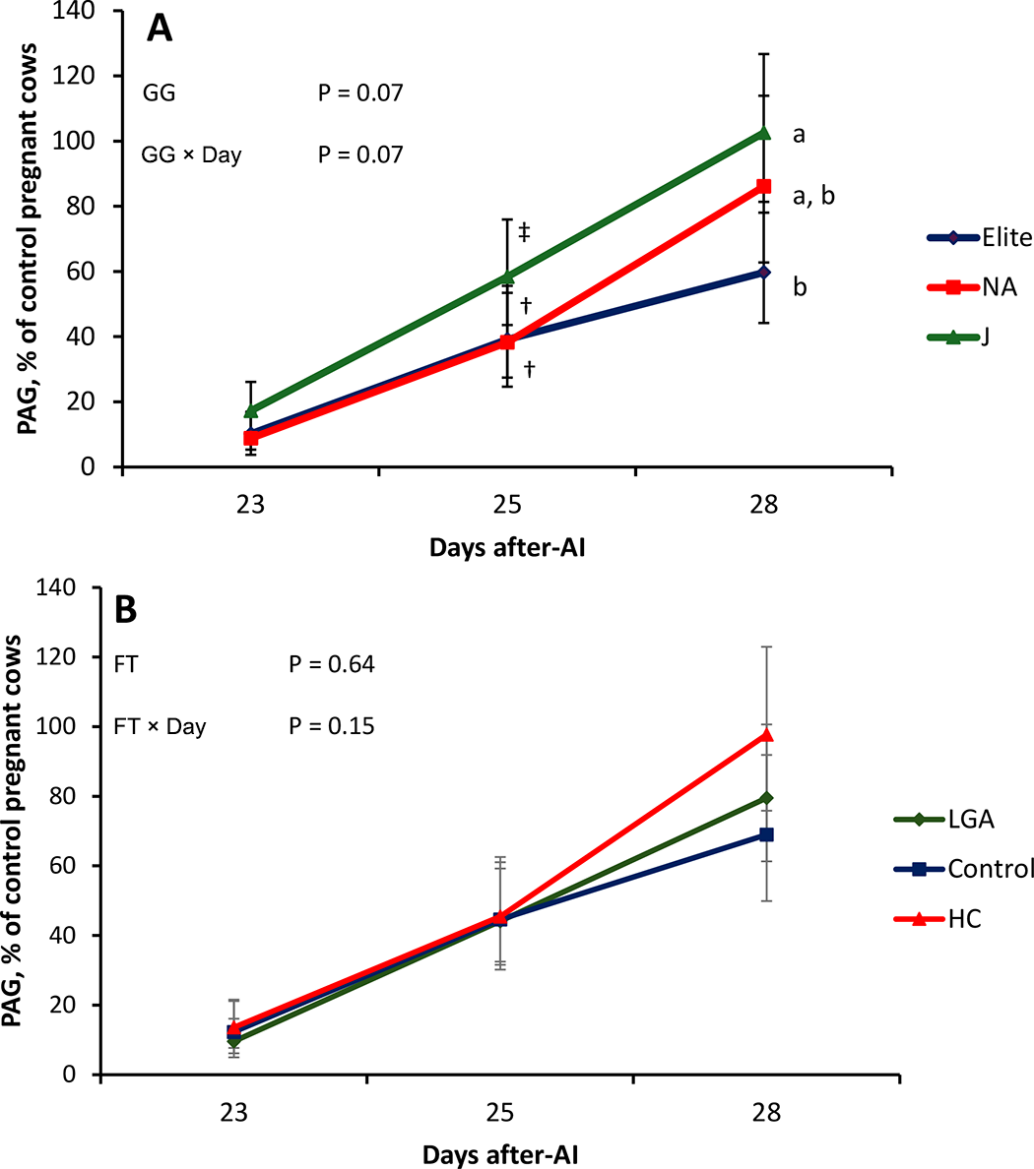
(A) The effect of genetic group (GG; Elite = ♦ [n = 20], NA = ■ [n = 13], Jersey = ▲ [n = 21]) on circulating plasma pregnancy-associated glycoprotein (PAG) concentrations from d 23 to d 28 post AI (expressed relative to d 35 pregnant cows [% PC]) for cows that maintained a successful pregnancy. All values are back-transformed LSM, and the error bars indicate 95% CI. There was evidence for both an overall effect of GG (*P* = 0.07) on mean plasma PAG concentrations and an interaction between GG and days post-AI (*P* = 0.07) on plasma PAG concentrations. Least squares means that do not share a common lowercase letter (a,b) letter on each day differ (*P* < 0.05). Least squares means that do not share a common symbol (†,‡) on each day differ (*P* > 0.05 and ≤0.10). (B) The effect of feed treatment (FT; LGA = ♦ [n = 20], control = ■ [n = 14], HC = ▲ [n = 20]) on circulating plasma PAG concentrations from d 23 to d 28 after AI (expressed relative to d 35 pregnant cows [% PC]) for cows that maintained a successful pregnancy. All values are back-transformed LSM, and the error bars indicate 95% CI. No effect of feed treatment (*P* = 0.64) or interaction between feed treatment and days after AI was observed (*P* = 0.15).

We found no effect of parity (*P* = 0.18) on mean circulating plasma PAG concentrations, but an interaction between parity and days after AI was observed (*P* = 0.002). This occurred because parity 1 cows had greater plasma concentrations on d 23 and 25 after AI (17.10% PC [9.9%, 26.8%], 56.1% PC [40.5%, 74.8%]) compared with parity 3+ cows (7.5% PC [3.6%, 13.3%], 34.4% PC [23.6%, 47.8%]), and had greater plasma PAG concentrations on d 28 after AI (96.2% PC [74.0%, 121.8%]) compared with parity 2 cows (61.1% PC [43.7%, 82.0%]). On d 28 after AI, parity 3+ cows had greater (*P* = 0.06) plasma PAG concentrations than parity 2 cows.

Between d 33 and 63 after AI, 9 cows (8.5%) were diagnosed as having had PL. Mean plasma PAG concentrations were greater (*P* < 0.0001) in cows that maintained a pregnancy (43.9% PC, [37.5%, 51.0%]) compared with cows that no evidence of pregnancy based on plasma PAG measurements in advance of ultrasound diagnosis on d 33 (0.13% PC, [0.01%, 0.48%]) and cows that were pregnant on d 33 but subsequently had PL by d 63 (15.2% PC, [8.03%, 25.6%]). An interaction (*P* < 0.0001) between cow pregnancy status group and days after AI was observed (Figure 2). The PREG cows had greater plasma PAG concentrations on d 23, 25, and 28 after AI (13.7% PC [10.3, 17.7], 51.2% PC [43.0, 60.4], 88.9% PC [76.9, 102.1]), compared with NOT PREG cows (0.07% PC [0.0005%, 0.38%], 0.13% PC [0.005%, 0.59%], 0.23% PC [0.02%, 0.86%]). Plasma PAG concentrations differed between PREG cows and PL cows on d 23 (*P* = 0.006), 25 (*P* = 0.0005), and 28 (*P* = 0.0001). Furthermore, NOT PREG cows had lower plasma PAG concentrations compared with PL cows on all sampling days.

**Figure 2:**
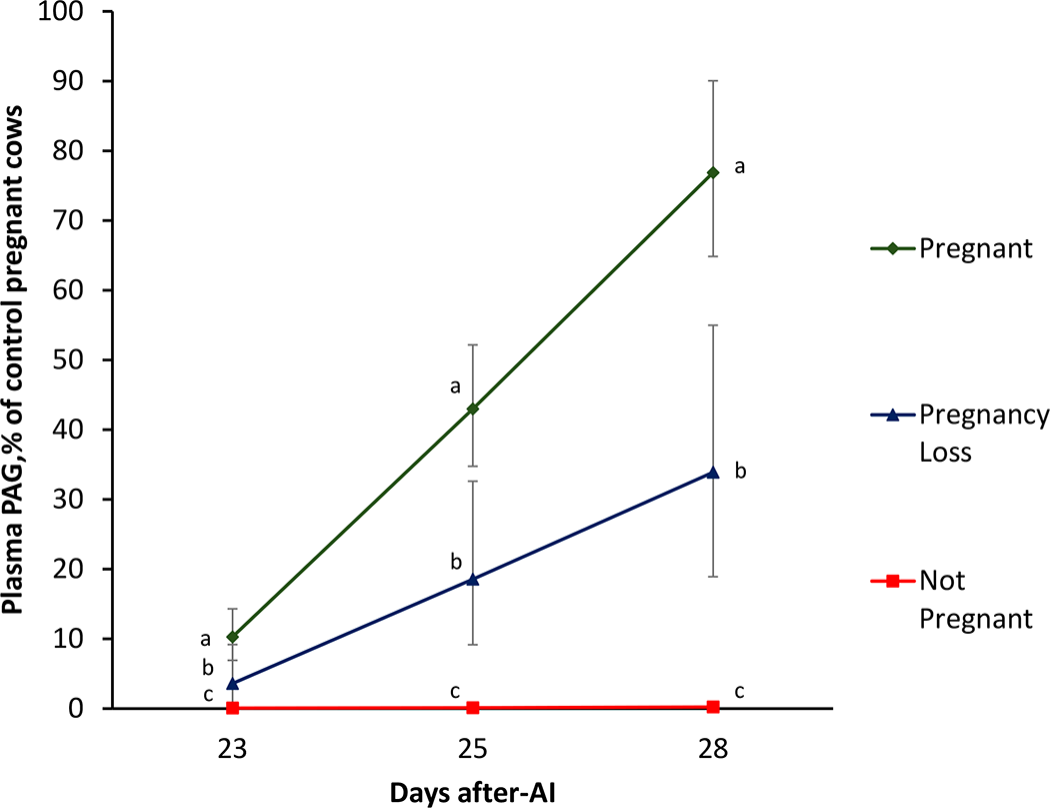
Plasma PAG concentrations for cows that maintained a successful pregnancy (pregnant ♦ [n = 54]), cows that had pregnancy loss between d 33 and 63 after AI based on ultrasound assessment (pregnancy loss ▲ [n = 9]) and cows that had no evidence of pregnancy based on plasma PAG concentrations (not pregnant ■ [n = 29]). The PAG concentrations are expressed relative to d 35 pregnant cows (% PC). Cow pregnancy status group was associated (*P* < 0.0001) with mean plasma PAG concentrations and an interaction between pregnancy status group and days post-AI (*P* < 0.0001) was observed. Values are back-transformed LSM, and the error bars indicate 95% CI. Least squares means that do not share a common lowercase letter (a–c) on each day differ (*P* < 0.05).

Pregnancy-associated glycoprotein assays can effectively diagnose pregnancy as early as d 23 in heifers, d 24 in beef cattle, and d 29 after AI in lactating dairy cows (Arnold et al., 2012; Oliveira Filho et al., 2020). Fetal sex, number of fetuses, fetal viability, stage of gestation, calf birth weight, dam genetic merit, phenotypic milk production, and parity of the dam have been previously reported to influence PAG concentrations in blood and milk (Patel et al., 1997; Mercadante et al., 2016; Doyle et al., 2025). In contrast to our original hypothesis, GG did not have a marked effect on circulating plasma PAG concentrations (Figure 1A). In a previous study, we observed an effect of dam genetic merit for fertility traits on milk PAG sample minus the negative control mean optical density (**S-N**) values between wk 5 and 6 of gestation, with greater PAG S-N values observed in cows with the greatest genetic merit for fertility traits (Doyle et al., 2025). This contrasts with the findings from the present study, where no effect of cow genetic background on plasma PAG concentrations was detected, but the studies differed in the time of sample collection and sample type (milk vs. plasma). A significant effect of dam breed was observed for mean plasma PAG concentration in the current study, with greater concentrations in JE compared with HF cows. This is consistent with a recent report that PAG levels in milk were greater in Jersey and Ayrshire cows compared with Holstein cows during early gestation (Albaaj et al., 2026).

Previous studies reported that cows with good genetic merit for fertility traits had more favorable fertility phenotypes, including reduced calving to conception interval and services per cow, greater circulating IGF-1, greater BCS (Cummins et al., 2012a), greater circulating estradiol and progesterone concentrations, greater CL volume (Cummins et al., 2012b; Moore et al., 2014b), improved uterine health, greater circulating glucose (Moore et al., 2014a), and a greater proportion of grade one blastocysts following superovulation (Moore et al., 2019) compared with cows with poor genetic merit for fertility traits. Using the same herd as the current study, no differences were detected in individual cow daily grass DMI or total DMI between the GG (O'Sullivan et al., 2019a). However, cows on the control FT had higher daily grass DMI than cows on the LGA or HC treatments, whereas total DMI was highest in cows on the HC treatment (O'Sullivan et al., 2019a). Elite cows had greater milk solids yield (O'Sullivan et al., 2019b), greater mean BCS, greater pregnancy per AI at first service, fewer services per cow, greater circulating concentrations of IGF-1 (O'Sullivan et al., 2020a), improved uterine health status, and greater circulating progesterone concentrations (Doyle et al., 2026) compared with NA cows. Despite these differences, pregnant Elite cows had similar circulating PAG concentrations as pregnant NA cows. It is well established that most PL occur before d 18 after estrus in grazing dairy cows (Berg et al., 2022; Crowe et al., 2025b). Hence, pregnancies that successfully progress through the stages of blastocyst formation, hatching, elongation, and maternal recognition of pregnancy within stage-specific time constraints have already overcome many of the critical milestones for pregnancy establishment. In the current study, all pregnancies had progressed through each of these milestones and achieved successful conceptus attachment (typically on or before d 21 after AI), and circulating PAG concentrations on d 23, 25, and 28 did not differ between Elite and NA cows. It is likely that effects on fertility performance due to differences in genetic merit for fertility traits are primarily due to pregnancy failures that occur before d 23. This is consistent with the present study, where no significant evidence of an effect of FT on PAG during early gestation was detected.

In the present study, the first blood sample was collected on d 23 after AI, which is 5 d earlier than the normal recommendation for pregnancy diagnosis using this PAG assay. In a retrospective study of seasonal-calving dairy cows, those that were pregnant at wk 5 but experienced embryo loss between wk 6 and 7 after AI had lower milk PAG S-N values compared with both cows that maintained their pregnancy and those that experienced PL at wk 8 or later (Doyle et al., 2025). Hence, PAG determination can be useful as an indicator of pregnancy status and embryo viability.

In the present study, PAG concentrations on d 23, 25, and 28 were greater in PREG cows compared with cows that had PL between d 33 and 63 of gestation. Cows with early embryonic loss were previously reported to have lesser concentrations of PAG on d 28 and 31 in blood (Pohler et al., 2016a,b). These same authors did not identify a relationship between the dam circulating PAG concentration and fetal development and concluded that the circulating PAG concentration was primarily an indicator of placental function (Pohler et al., 2014). Measurement of PAG therefore could aid identification of pregnancies at risk of loss before ultrasound detection. This could enable more frequent monitoring and earlier intervention, although further research is required to establish accurate threshold values for predicting PL. An important limitation of this pilot-scale study was the small number of cows that had PL, which reduced statistical power and constrained detection of interaction effects. Moreover, these results are associative and should not be interpreted as predictive or diagnostic without further validation in larger, prospective studies.

In conclusion, JE cows had greater plasma PAG concentrations during early gestation than HF cows, but Elite and NA HF cows did not differ, and no effect of FT was observed. Improved reproductive performance in cows with better genetic merit for fertility traits, therefore, was not associated with differences in PAG during the early stages of pregnancy. This suggests that fertility differences between cows with divergent genetic merit for fertility traits manifests before conceptus attachment when the incidence of PL is greatest. The potential role of early PAG determination (on or before d 28) to identify at-risk pregnancies and predict pregnancy losses warrants further investigation.
